# Three and six grams supplementation of d-aspartic acid in resistance trained men

**DOI:** 10.1186/s12970-015-0078-7

**Published:** 2015-04-01

**Authors:** Geoffrey W Melville, Jason C Siegler, Paul WM Marshall

**Affiliations:** School of Science & Health, University of Western Sydney, Campbelltown Campus, Locked Bag 1797, Penrith, NSW 2751 Australia

**Keywords:** D-aspartic acid, Resistance training, Testosterone, Estradiol, SHBG

## Abstract

**Background:**

Although abundant research has investigated the hormonal effects of d-aspartic acid in rat models, to date there is limited research on humans. Previous research has demonstrated increased total testosterone levels in sedentary men and no significant changes in hormonal levels in resistance trained men. It was hypothesised that a higher dosage may be required for experienced lifters, thus this study investigated the effects of two different dosages of d-aspartic acid on basal hormonal levels in resistance trained men and explored responsiveness to d-aspartic acid based on initial testosterone levels.

**Methods:**

Twenty-four males, with a minimum of two years’ experience in resistance training, (age, 24.5 ± 3.2 y; training experience, 3.4 ± 1.4 y; height, 178.5 ± 6.5 cm; weight, 84.7 ± 7.2 kg; bench press 1-RM, 105.3 ± 15.2 kg) were randomised into one of three groups: 6 g.d^−1^ plain flour (D0); 3 g.d^−1^ of d-aspartic acid (D3); and 6 g.d^−1^ of d-aspartic acid (D6). Participants performed a two-week washout period, training four days per week. This continued through the experimental period (14 days), with participants consuming the supplement in the morning. Serum was analysed for levels of testosterone, estradiol, sex hormone binding globulin, albumin and free testosterone was determined by calculation.

**Results:**

D-aspartic acid supplementation revealed no main effect for group in: estradiol; sex-hormone-binding-globulin; and albumin. Total testosterone was significantly reduced in D6 (P = 0.03). Analysis of free testosterone showed that D6 was significantly reduced as compared to D0 (P = 0.005), but not significantly different to D3. Analysis did not reveal any significant differences between D3 and D0. No significant correlation between initial total testosterone levels and responsiveness to d-aspartic acid was observed (r = 0.10, P = 0.70).

**Conclusions:**

The present study demonstrated that a daily dose of six grams of d-aspartic acid decreased levels of total testosterone and free testosterone (D6), without any concurrent change in other hormones measured. Three grams of d-aspartic acid had no significant effect on either testosterone markers. It is currently unknown what effect this reduction in testosterone will have on strength and hypertrophy gains.

## Background

The anabolic hormone testosterone is considered to be a key determinant of training induced improvements in hypertrophy and strength. Circulating testosterone increases other anabolic hormones and directly interacts with androgen receptors and satellite cells, causing a cascade of events leading to protein synthesis and muscle growth [1,2]. Research has previously demonstrated correlations between testosterone levels and training related strength gains [3,4]. Furthermore exogenous elevation of testosterone to supraphysiological levels, via administration of anabolic steroids has been shown to drastically improve strength and hypertrophy [5]. Currently it is unknown whether boosting testosterone levels within normal physiological levels (mid-range to upper-range) will have a significant effect on strength and hypertrophy. Nonetheless, the supplement industry is endorsing testosterone boosters to improve training related gains. D-aspartic acid is currently recommended as a viable product to significantly raise testosterone, however research in humans only supports this recommendation in untrained men with below average testosterone levels. Moreover there is no information about the effect of different doses of d-aspartic acid on testosterone levels in humans.

Aspartic acid (C_4_H_7_NO_4_) is an α-amino acid which is known to exist in two isoforms, l-aspartic acid and d-aspartic acid. (2R)-2-aminobutanedioic acid or d-aspartic acid (DAA), previously believed to be exclusive to brain tissue in octopus, squid and cuttlefish, has more recently been shown to exist in mammals [6]. Free DAA is found in tissues and cells related to the central nervous and endocrine systems [7,8]. DAA is believed to stimulate the production and release of testosterone through multiple pathways of the hypothalamic-pituitary-gonadal (HPG) axis. It has been shown to increase steroidogenic acute regulatory protein (StAR) gene expression in rat Leydig cells [9]. StAR is a key regulator for the transport of cholesterol from outside the mitochondrial membrane to the inner membrane [7]. By increasing levels of StAR DAA may indirectly increase testosterone, as the transportation of cholesterol is believed to be the rate limiting step in the production of testosterone [7]. *In vitro* rats studies demonstrated that DAA increased levels of testosterone, luteinizing hormone, progesterone [6] and growth hormone [10]. This is believed to occur due to the accumulation of DAA in the anterior pituitary and testes [10]. Additional *in vitro* studies on isolated rat testes [6] and Leydig cells [11] indicate that DAA increased the rate of testosterone synthesis in a dose dependent manner. In these animals the maximal effective dose of DAA, which elicited the greatest hormonal response (LH, testosterone and progesterone), was 1 μmol.g^−1^ [6]. In humans the effects of different dosages of DAA on basal testosterone is unclear.

To date only two studies on DAA supplementation have been conducted on humans. Topo et al. [12] demonstrated that after 12 days of supplementation (3.12 g.d^−1^), levels of testosterone were significantly increased by 42% (4.5–6.4 ng.ml^−1^). They recruited a cohort of healthy sedentary male IVF patients (27–37 years), with low initial testosterone levels (~4.55 ng.ml^−1^). Contrastingly Willoughby and Leutholtz, reported that after 29 days of supplementation (3 g.d^−1^) and resistance training, levels of total testosterone and free testosterone were not significantly altered. In this study resistance trained men (age: 22.8 ± 4.67 years old; training age: > 1 year) were recruited and this cohort exhibited higher initial testosterone levels (~7.96 ng.ml^−1^) [13]. The difference in outcome between these two studies may in part be explained by training status and accompanying basal testosterone levels. Basal testosterone levels of RT men range from approximately 5.8–8.6 ng.ml^−1^ (20–30 nmol.l^−1^), [4,14] and untrained men range from about 4.9–6.6 ng.ml^−1^ (17–23 nmol.l^−1^) [15-17]. Furthermore current research has only explored one dose response of DAA, 3 g.d^−1^ [12,13], hence the maximum effective dose for humans is yet to be determined.

Supplement companies are currently recommending three grams of DAA once to twice a day, and these recommendations have been drawn from the only dosage studied in humans (3 g.d^−1^). It is reasonable to believe that in RT males, a higher dose may be required to further increase testosterone levels. As such the primary aim of this study was to evaluate the effects of two doses of d-aspartic acid (3 g and 6 g) on basal testosterone levels in resistance trained men. A secondary aim was to establish if a relationship exists between initial testosterone levels and responsiveness to DAA. It was hypothesised that; (a) testosterone levels would be unchanged in the 3 g group; (b) testosterone levels would be increased in the 6 g group; and (c) lower initial testosterone levels would correspond with an increased responsiveness to DAA.

## Methods

### Subjects

The institutional review board approved the study and participants provided written informed consent prior to testing and participation. A total of twenty-four participants from the local area completed this study (Table 1). To be eligible participants had to be: male; aged 18–36; have no acute or chronic medical conditions; have the ability to bench press 100% bodyweight; and had been performing regular resistance training exercise for at least three days per week for the previous two years. None of the participants were supplementing their diet with any ergogenic or testosterone booting supplements prior to testing. All participants provided written consent and completed a medical history check. The study was approved by the University of Western Sydney human research ethics committee, and carried out in accordance with the declaration of Helsinki.

**Table 1:** **Participant demographics**

	**Placebo (** ***n*** **= 8)**	**3 g.d** ^**−1**^ **(** ***n*** **= 8)**	**6 g.d** ^**−1**^ **(** ***n*** **= 8)**
Age (years)	24.24 ± 2.26	23.16 ± 2.16	26.06 ± 4.26
Training age, (years)	2.94 ± 0.78	3.25 ± 1.04	4.00 ± 1.91
Height (m)	1.84 ± 0.03	1.74 ± 0.07	1.78 ± 0.06
Body Mass (kg)	89.41 ± 3.59	79.50 ± 6.07	85.12 ± 7.95
1 RM Bench (kg)	111.56 ± 15.17	97.50 ± 12.82	106.86 ± 15.74

Data are mean ± SD.

### Experimental approach to the problem

**Figure 1:**
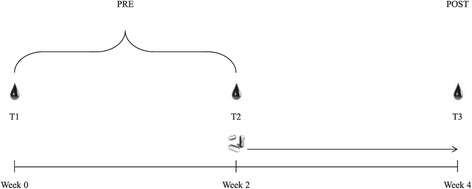
**Timeline of the study.** After completion of T1, subjects began training four days per week. Daily supplementation commenced after T2 (). T1-3 included fasted blood draws ().

This was a randomised, double-blinded, and placebo-controlled design to examine the effects of d-aspartic acid supplementation on basal testosterone levels following a two week supplementation protocol. Participants were assigned to one of three experimental groups: placebo (D0), three grams of DAA (D3) and six grams of DAA (D6). All participants consumed 10 opaque capsules each morning with breakfast for two weeks. They contained either: six grams of flour (D0, n = 8); a mixture of three grams each of flour and DAA (D3, n = 8); or six grams of DAA (D6, n = 8). Participants were randomly allocated to treatment groups following a block randomisation procedure based on a computer-generated list of random numbers. Placebo, mixed and supplement were provided in identical opaque capsules to improve blinding. Group allocation was managed by a technical officer, whilst investigators were kept blind to group assignment throughout the intervention. All participants followed an upper/lower body split resistance training program for a full month, with the initial two weeks of training (washout period) performed without supplementation (Figure 1). Three timepoints were used to obtain testing data: T1, T2 and T3 (Figure 1).

### Experimental procedures

Testing sessions consisted of a fasted blood draw, then 1-RM bench press evaluation. Initial baseline blood measures were taken at two timepoints (T1 & T2) and averaged to ensure accuracy in baseline assessment of these markers (Figure 1). After T1 prescribed training commenced for four weeks. After testing session T2 daily supplementation begun with training continuing as before. Post-measures were taken after these last two weeks of training and supplementation, at the end of week 4 (Figure 1). The supplemental period of two weeks was chosen as this has been previously shown to be a sufficient time period to see a change in total testosterone levels [12].

### 1-RM testing

Bench press dynamic strength one repetition max (1-RM) was measured before the standardisation period (T1), beginning of experimental period (T2) and post experiment period (T3) (Figure 1), as part of eligibility testing. Correct form included depth to the level of the chest, with feet not leaving the floor, and the backside not leaving the bench at any point during the repetition. The protocol for 1-RM testing involved one warm up set of 10 reps at approximately 50% of their estimated 1-RM, followed by two more warm ups at approximately 70% and 80% with only 1–2 reps. After the warm ups participants attempted 1-RMs with incrementally increasing weight. The weight achieved prior to the failed attempt was recorded as the 1-RM. A participant’s 1-RM was achieved within five attempts and adequate rest between attempts was adhered to (3–5 mins) [18].

### Fasted blood draws

All blood draws were obtained via venepuncture of the antecubital vein after a 12 hour fast. Participants were also instructed to avoid strenuous exercise and alcohol consumption the day before the draw. Blood draws were conducted by a trained phlebotomist and subsequent draws were planned for the same time of morning (7:00–10:00 am) for each particular participant, to prevent any effect of diurnal variation. Whole blood was collected using serum separator tubes (SST™ II Advance, BD Vacutainer®). They were then allowed to clot for 45 minutes and centrifuged using a fixed angle rotor centrifuge: ADAMS® Compact II Centrifuge, V:227 (Becton Dickinson & Co) (828 × g, at 2700 rpm) for 15 minutes in an air conditioned room (19°C). Serum was aliquoted and stored at −80°C until analysis (Douglas Hanly Moir Pathology, Macquarie Park, NSW, Australia). Single analysis of serum was conducted for total testosterone, estradiol, sex-hormone-binding-globulin (SHBG) and albumin. Testosterone and SHBG was measured via electrochemiluminescent (ECL) immunoassay, on a Roche E170 system (Roche Diagnostics). Albumin was measured via bromocresol green (BCG) succinate buffer method, on an Abbott 16000. Estradiol was measured via chemiluminescent microparticle immunoassay on an Abbott i2000. Free testosterone was calculated from total testosterone, SHBG and albumin.

### Training standardisation

Participants trained for four days per week over a one month period. The prescribed training for each exercise consisted of four sets of a repetition maximum range of 8–10. If the repetition range wasn’t met, participants were asked to lower or raise the weight in the next session. Exercises during the upper body session were: barbell bench press; overhand pulldown; barbell overhead press and underhand pulldown. The lower body session consisted of: back squat; good morning; leg extensions; and straight leg calf raises. Adherence was monitored via training diaries and supervised sessions (minimum 1 × per week).

### Dietary intake

Participants were asked to control their diet, by avoiding any major changes throughout the study duration. To monitor their diet they were asked to weigh and recorded their food intake for three days each of the first and last week; two training days and one non-training day. These three days were averaged to get a daily mean for week one and four. The food diaries were entered into CalorieKing (Australian Edition 4.0), then analysed for caloric and macronutrient daily intakes (protein, carbohydrates and fats) and normalised to bodyweight.

### Statistical analysis

Analyses were conducted using IBM SPSS Statistics for Windows version 21.0 (Armonk, NY: IBM Corp), and the level of significance was set at P < 0.05. Data are shown as mean ± S.D. The distribution was tested for normality using the Kolmogorov-Smirnov test. Paired sample statistics were run on total testosterone (TT), free testosterone (FT), estradiol (E_2_), sex-hormone-binding-globulin (SHBG), and albumin (ALB) to determine the stability of these blood measures over the standardisation period. As these measures were found to be unchanged they were each computed (averaged) into one baseline measure. Univariate analysis of the absolute change scores: $\varDelta =\left(T3-\frac{T1+T2}{2}\right)$was conducted, with the baseline scores: $\mathrm{P}\mathrm{R}\mathrm{E}=\left(\frac{T1+T2}{2}\right)$as covariates (Figure 1). Pairwise comparisons with Bonferroni correction were performed if a group effect was observed. To explore the responsiveness of the supplement, linear regression analysis was conducted on the baseline and change scores of TT and FT, of the experimental groups (*n* = 16).

## Results

**Figure 2:**
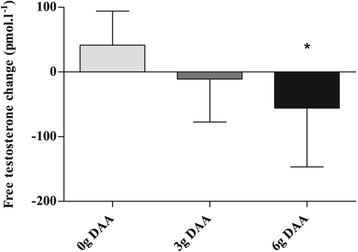
**The absolute change of free testosterone.** *statistically significant (P < 0.05).

Analysis of the POST values revealed no main effect for group with E_2_ (P = 0.47), SHBG (P = 0.07) and ALB (P = 0.32). Post values of D6 TT were significantly reduced (~12.5%) as compared to the pre values (P = 0.03; 5.9 to 5.1 ng.ml^−1^). FT in group D6 was significantly decreased (429.1 to 363.4 pmol.l^−1^) as compared to D0 (439.6 to 480.9 pmol.l^−1^) (P = 0.005) but not D3 (534.9 to 524.3 pmol.l^−1^) (P = 0.06) (Figure 2). Diet analysis revealed no significant changes in macronutrient (CHO: P = 0.74; PRO: P = 0.99; FAT: P = 0.54) and caloric intakes (P = 0.64) during the study. Regression analysis revealed no significant correlation between baseline total testosterone levels and total testosterone change (r = 0.10, P = 0.70), and no significant correlation between baseline free testosterone and free testosterone change (r = 0.32, P = 0.23).

## Discussion

The primary findings of the current study were, 1) resistance trained men consuming six grams of d-aspartic acid daily demonstrated significant reductions in total and free testosterone after 14 days of d-aspartic acid supplementation, and 2) the responsiveness to d-aspartic acid supplementation was unaffected by initial testosterone levels (total or free) in resistance trained men.

Our results demonstrate that in resistance trained men three grams daily of d-aspartic acid had no significant effect on total testosterone, estradiol, sex-hormone-binding-globulin, and albumin. This is contrary to the evidence provided by Topo et al. [12], where the cohort consumed the same dose over 12 days and reported elevated total testosterone levels (~42%). Baseline testosterone levels of the current study were higher than values found in Topo et al. [12] (6.3 and 4.5 ng.ml^−1^ respectively), presumably because the cohort in the Topo et al. study were sedentary [12]. In resistance training literature, total testosterone levels range from 5.8–8.6 ng.ml^−1^ [4,14] for trained individuals and 4.9–6.6 ng.ml^−1^ for untrained [15-17]. The increase in testosterone observed in Topo et al. [12] was likely due to the fact that testosterone levels were low enough for d-aspartic acid to have an effect. In comparison our results in the D3 group mirror the results seen in the study by Willoughby & Leutholtz [13], where the total testosterone levels fall within levels observed in resistance trained males [4,14].

It was observed in the six gram group that total testosterone was significantly reduced from baseline by ~12.5%, with a parallel decrease in free testosterone ~15.3% (see Table 2). Previous research has demonstrated that in resistance trained men, free testosterone can increase due to training [19]. A reduction in calculated free testosterone in this study is due to a reduction in total testosterone, an increase in the binding proteins or a combination of the two occurring. Within the context of increasing total testosterone a maximum effective dosage (MED) is observed in rat studies [6]. At the higher dosages there were significantly increased accumulation of d-aspartic acid observed in the pituitary and testes [6]. A dose response increase in total testosterone was observed until 1 μmol.g^−1^. Each increase in dose past 1 μmol.g^−1^ the rise in testosterone was reduced [6]. It could be theorised that 6 g.d^−1^ may be affecting negative feedback mechanisms of the HPG axis, thus reducing pituitary initiated production of luteinizing hormone and in turn testosterone levels. Furthermore d-aspartic acid could also be over-accumulating within the testes. This may be creating a disruptive effect on the mobilisation of cholesterol from the outer membrane to the inner [7], which would attenuate testosterone production. As this was the first study to administer a six gram dosage of d-aspartic acid, these mechanisms can only be speculated due to the lack of data available on the utilisation of d-aspartic acid in humans.

**Table 2:** **PRE (Baseline), POST (T3), and Change Scores (Δ) of hormonal markers**

	Total Testosterone (ng.ml^−1^)		
Time	Placebo	3 g.d^−1^	6 g g.d^−1^
PRE	6.03 ± 1.48	6.95 ± 1.44	5.85 ± 1.10
POST	6.07 ± 1.35	6.91 ± 1.71	5.12 ± 1.16
Δ	0.05 ± 0.80	−0.03 ± 0.68	−0.74 ± 0.95*
	Free Testosterone (pmol.l^−1^)		
	Placebo	3 g.d^−1^	6 g g.d^−1^
PRE	439.62 ± 132.64	534.88 ± 127.65	429.13 ± 93.98
POST	480.87 ± 133.48	524.25 ± 101.67	363.38 ± 78.09
Δ	41.25 ± 52.48	−10.63 ± 66.31	−65.75 ± 79.25*
	Estradiol (pmol.l^−1^)		
	Placebo	3 g.d^−1^	6 g g.d^−1^
PRE	118.50 ± 20.91	117.56 ± 30.58	107.50 ± 24.22
POST	125.12 ± 23.88	112.5 ± 34.51	104.75 ± 34.03
Δ	6.63 ± 14.94	−5.06 ± 19.52	−2.75 ± 23.46
	SHBG Pre (nmol.l^−1^)		
	Placebo	3 g.d^−1^	6 g g.d^−1^
PRE	34.56 ± 16.55	32.56 ± 10.72	33.56 ± 11.82
POST	30.38 ± 12.39	32.88 ± 12.53	33.75 ± 10.98
Δ	−4.19 ± 5.90	0.31 ± 4.29	0.19 ± 1.46
	Albumin (g.l^−1^)		
	Placebo	3 g.d^−1^	6 g g.d^−1^
PRE	46.38 ± 2.08	45.06 ± 2.60	45.50 ± 1.49
POST	44.75 ± 1.67	45.00 ± 2.33	45.50 ± 2.56
Δ	−1.63 ± 1.33	−0.06 ± 1.82	0.00 ± 2.35

Data is presented as: mean ± standard deviation.

*statistically significant (P < 0.05).

PRE values are an average of T1 and T2.

The reductions in testosterone observed in this study are important to consider, owing to the negative impact it could have on training gains within this population. Resistance trained men have higher levels of strength and hypertrophy compared to novice trainers and also exhibit higher basal testosterone levels [4,13-17], which suggest a link between basal total testosterone levels and training related gains. A decrease in total testosterone with a concurrent decrease in free testosterone could reduce the likelihood of interaction with androgen receptors in muscles and nerves, which would reduce the speed of testosterone initiated muscle protein synthesis [1]. Over time this could translate into reduced training gains. Conversely, alterations of testosterone within normal physiological ranges may not be clinically significant. Research indicates that when total testosterone levels are observed outside of normal healthy ranges (4.9-8.6 ng.ml^−1^) it affects strength and hypertrophy. In the case of hypogonadism where testosterone levels are low this negatively affects strength and hypertrophy, and with the use of steroids a positive affect is seen [5,20]. The changes observed in the current study reflect minor alterations with respect to normal physiological ranges. It is currently unknown if these fluctuations are detrimental to training gains.

A potential limitation of this research may be the study length. The short term nature of a two week supplementation period will answer only acute hypotheses. The observed reduction in testosterone may rebound, or even decrease further and a longer term training study would be able to better explain the effects of this supplement. Moreover it would be able to delineate changes in strength and or hypertrophy, and observe whether d-aspartic acid affects training related gains positively or negatively.

## Conclusion

Many testosterone boosting supplements are commercially available without sufficient research to support their efficacy. The present study has demonstrated that 3 g.d^−1^ of d-aspartic acid was inadequate to affect any hormonal markers and that 6 g.d^−1^ significantly reduced total testosterone and free testosterone levels, with no concurrent change in other hormones tested. It is currently unknown if any negative consequences of this reduction, with respect to strength and hypertrophy will occur over time. The need for longer-duration research utilising six grams of d-aspartic acid is clear. Future research should explore supplementation of 6 g.d^−1^ over a longer period and observe any correlations between basal testosterone levels and changes in hypertrophy and strength.
